# A tale of two plasmids: contributions of plasmid associated phenotypes to epidemiological success among *Shigella*

**DOI:** 10.1098/rspb.2022.0581

**Published:** 2022-08-10

**Authors:** P. Malaka De Silva, George E. Stenhouse, Grace A. Blackwell, Rebecca J. Bengtsson, Claire Jenkins, James P. J. Hall, Kate S. Baker

**Affiliations:** ^1^ Department of Clinical Infection, Microbiology and Immunology, Institute of Infection, Veterinary and Ecological Sciences, University of Liverpool, Liverpool, UK; ^2^ Department of Evolution, Ecology and Behaviour, Institute of Infection, Veterinary and Ecological Sciences, University of Liverpool, Liverpool, UK; ^3^ EMBL-EBI, Wellcome Sanger Institute, Wellcome Genome Campus, Hinxton, Cambridgeshire, CB101SA, UK; ^4^ Department of Parasites and Microbes, Wellcome Sanger Institute, Wellcome Genome Campus, Hinxton, Cambridgeshire, CB101SA, UK; ^5^ Gastro and Food Safety (One Health) Division, UK Health Security Agency (UKHSA), Colindale, London, UK

**Keywords:** conjugation, plasmid fitness cost, antimicrobial resistance, bacterial SOS response

## Abstract

Dissemination of antimicrobial resistance (AMR) genes by horizontal gene transfer (HGT) mediated through plasmids is a major global concern. Genomic epidemiology studies have shown varying success of different AMR plasmids during outbreaks, but the underlying reasons for these differences are unclear. Here, we investigated two *Shigella* plasmids (pKSR100 and pAPR100) that circulated in the same transmission network but had starkly contrasting epidemiological outcomes to identify plasmid features that may have contributed to the differences. We used plasmid comparative genomics to reveal divergence between the two plasmids in genes encoding AMR, SOS response alleviation and conjugation. Experimental analyses revealed that these genomic differences corresponded with reduced conjugation efficiencies for the epidemiologically successful pKSR100, but more extensive AMR, reduced fitness costs, and a reduced SOS response in the presence of antimicrobials, compared with the less successful pAPR100. The discrepant phenotypes between the two plasmids are consistent with the hypothesis that plasmid-associated phenotypes contribute to determining the epidemiological outcome of AMR HGT and suggest that phenotypes relevant in responding to antimicrobial pressure and fitness impact may be more important than those around conjugation in this setting. Plasmid phenotypes could thus be valuable tools in conjunction with genomic epidemiology for predicting AMR dissemination.

## 1. Introduction

Antimicrobial resistance (AMR) is a pressing global public health crisis. Bacterial pathogens become resistant to antimicrobials through either chromosomal mutations or by acquiring new AMR determinants through horizontal gene transfer (HGT) of mobile genetic elements (MGEs), such as plasmids [1,2]. HGT plays an important part in the dissemination of AMR genes, evidenced by multiple reports of emergence of similar AMR genes from different locations around the world. For example, plasmid-mediated colistin resistance conferred by the *mcr-1* gene was first reported in 2015 in an isolate from 2011 in China, and was subsequently identified across five continents [3,4] in multiple species of Enterobacteriaceae by virtue of its being carried on a plasmid capable of inhabiting multiple hosts after it was first mobilized by a composite transposon [5]. Similarly, *Klebsiella pneumoniae* carbapenemases (KPCs), which were originally observed in the USA in 1996 [6,7], and CTX-M extended spectrum beta lactamases (ESBL), which were thought to be mobilized from the chromosome of *Kluyvera* spp., have been reported in multiple geographical regions as a result of AMR plasmid dissemination [8–11]. Owing to the rapid and extensive dissemination of AMR genes through HGT, it is critical to understand how different plasmid ‘vehicles’ affect the behaviour and spread of AMR genes.

Headway has been made towards understanding AMR epidemiology with reference to the specific plasmids that carry AMR determinants for a number of pathogen–plasmid combinations, largely from hospital settings [12–16]. *In vitro* work as well as modelling studies—in some cases supported by epidemiological data—suggest that the phenotypes of plasmids, such as plasmid fitness costs, resistance gene profile and other plasmid-conferred traits, may have a role in driving transmission and persistence of AMR across bacterial host populations [17–22]. Recent studies have attempted to associate non-AMR phenotypes such as conjugation with pathogen and plasmid epidemiology, including in clinical settings [12,13,23]. However, the drivers of AMR-HGT in non-hospital associated pathogens are likely to be distinct owing to the environmental differences (e.g. in antimicrobial pressures and transmission routes) and the comparatively under-observed bacterial populations.

Tracking plasmid-mediated AMR emergence in the community therefore requires a well-surveyed and characterized pathogen population. The Gram-negative diarrhoeal pathogen *Shigella* provides a highly observable community infection because infection is almost always symptomatic, the disease is reportable and there is no substantial animal or environmental reservoir (with the exception of sporadically reported cases in non-human primates [24,25]). Using genomic epidemiology, we recently investigated the emergence of AMR in the relatively closed transmission network of *Shigella* infections among men who have sex with men (MSM) in the United Kingdom [26,27]. The selective pressure caused by antimicrobial treatment of sexually transmitted infections (STIs) such as syphilis and gonorrhoea (common co-infections among shigellosis affected MSM [26,28]) led to the global emergence of a sublineage of *Shigella flexneri* 3a following the acquisition of plasmid pKSR100, encoding azithromycin resistance [26]. This sustained selection pressure subsequently led to the convergent acquisition of multiple azithromycin resistance plasmids in multiple other *Shigella* sublineages, directly triggering and intensifying epidemic waves of shigellosis [27,29]. The acquisition of different azithromycin resistance plasmids by these different sublineages provides us with an epidemiologically grounded model to test why some plasmids proliferate in a population, and why some do not.

The two IncFII plasmids under study here derive from a cross-sectional analysis of UK *Shigella* strains detected during routine surveillance between 2008 and 2014. During this period, we demonstrated that *S. flexneri* 2a emerged within the MSM community (during a cross-sectional study of 179 isolates) as two azithromycin resistant sublineages with markedly different epidemiology [27]. The ‘minor’ sublineage was estimated to have emerged earlier (most recent common ancestor (MRCA) in 1996) but only seven cases were observed in the cross section during the study period, whereas the comparatively recent (MRCA 2011) persistent or ‘major’ sublineage, caused 49 cases in the cross section during the study period, and disseminated internationally [27,29,30]. Although both sublineages were azithromycin resistant, the plasmids conferring azithromycin resistance varied. The successful ‘major’ sublineage carried pKSR100 [27], a plasmid which has continued to spread globally throughout shigellae, while the other ‘minor’ sublineage carried azithromycin resistance on a different plasmid (herein termed pAPR100) which has failed to mirror the epidemiological success of pKSR100. Furthermore, pKSR100 has continued to acquire novel AMR genes including a multidrug resistance (MDR) integron [27] and, more recently, ESBL genes [31]. As both pKSR100 and pAPR100 plasmids conferred the critical azithromycin resistance phenotype, we hypothesized that non-azithromycin resistance related plasmid phenotypes of these co-circulating plasmids may have contributed to their disparate epidemiological outcomes.

Thus here, we use these two plasmids to examine which phenotypes conferred on the bacterial host by the plasmid associate with the emergence of AMR in a community-transmitting obligate pathogen. We extend the genomic epidemiological evidence for the respective global distribution of the plasmids and use a comparative genomics-guided approach to determine the discrepant phenotypes between these two co-circulating plasmids with markedly different epidemiological outcomes.

## 2. Material and methods

### (a) Plasmid comparison and comparative genomics

Full plasmid sequences were extracted from genome sequences from a previous study [27]. Specifically, for pKSR100 (now deposited in NCBI under accession CP090161) the isolate corresponding to accession number ERR1364116 was used and for pAPR100 (now deposited in NCBI under accession CP090162) the contiguous sequence 24 from the isolate corresponding with accession number ERR1364014 was used.

A detailed version of the comparative genomics methods is available in the electronic supplementary material. Briefly, extracted genomic sequences of the plasmids were annotated using RAST server [32] and regions of similarity were identified as BLAST percentage identity cut-off of 95% or above. Roary v. 3.11.2 [33] was then used to examine the unique regions of the two plasmids (electronic supplementary material, file S2). NCBI non-redundant database and 661 K COBS data structure were used to assess the global distribution of the two plasmids.

### (b) Bacterial conjugation

All bacterial strains used in conjugation experiments were grown in TSB overnight and diluted 1 : 100 into fresh media and grown for 3 h before preparing the conjugation mixture. Donor and recipient strains were mixed 1 : 1 in a final volume in 500 µl for the conjugation experiments in liquid media and incubated shaking at 215 r.p.m. at 37°C for 75 min. Conjugation mixtures were serially diluted and plated on selective media to distinguish between donors, recipients and transconjugants every 15 min and the resulting colony forming units were counted to enumerate each. Conjugation mixtures were prepared as described above for the conjugation experiments on solid media and 10 µl of the conjugation mixture was spotted onto a sterile nitrocellulose filter paper placed on an agar plate and incubated at 37°C without shaking for 5 h. Conjugation mixtures present on the filter papers were submerged in 500 µl of sterile PBS, agitated by vortexing, serially diluted and plated as above every hour for CFU measurements. Conjugation efficiencies (CEs) for four biological replicates were calculated as previously described [34] using the following equation:

$$\eta =\frac{T}{DR\;\Delta t}$$

where *η* = conjugation efficiency (CE) measured in ml cell^−1^ h^−1^, *T* = transconjugants, *D* = donors, *R* = recipients and Δ*t* = total conjugation time.

### (c) SOS response measurements during conjugation and ciprofloxacin exposure

SOS induction during both conjugation and exposure to ciprofloxacin was measured by using GFP expression as a proxy from a Pint-gfp fusion reporter plasmid p9092 (kindly gifted by Mazel and co-workers [35]) where the expression of the integrase promoter Pint is a strong signal of SOS induction [35]. Briefly, the strains of interest were transformed with p9092 where the GFP expressing cells were counted using a Bio-Rad ZE5 Cell Analyzer flow cytometer under the relevant test conditions and the proportion of GFP expressing cells were calculated for each instance. Specific details for each of the experiments are available in the electronic supplementary material.

### (d) Bacterial growth curves and fitness

Transconjugant *Escherichia coli* strains (with constitutive GFP expression) or *Shigella sonnei* 216 strains, carrying pKSR100 or pAPR100 from conjugation experiments were frozen in 25% glycerol and used as the source freezer stock for the growth curves. Pre-cultures for the bacterial growth curves were grown in M9 medium without azithromycin selection overnight incubating at 37°C with 215 r.p.m. shaking and diluted 1 : 100 into fresh M9 media adjusting all the cultures to the lowest OD600 value. The cultures were then distributed into a sterile 96-well plate (Greiner Bio One, UK) with a moisture barrier seal (4titude, UK) and incubated at 37°C with shaking in a Synergy H1 multi-mode plate reader taking optical density readings at 600 nm every 15 min. The resulting values were plotted using the R package Growthcurver [36] to determine the area under the curve (AUC) for each growth curve. Average values of the technical replicates for AUC of each of the plasmid carrying strains were normalized using the average AUC value of the technical replicates of plasmid-free *E. coli* and *S. sonnei* 216 strains to obtain relative fitness of each of the transconjugant *E. coli* and *S. sonnei* 216 strains for three biological replicates.

### (e) Minimum inhibitory concentration measurements

Minimum inhibitory concentration (MIC) measurements were carried out using Liofilchem MIC test strips (Liofilchem, Italy) following manufacturer's guidelines. Bacterial inocula for the MIC testing were prepared following the EUCAST guidelines for broth microdilution testing breakpoint table (https://www.eucast.org/fileadmin/src/media/PDFs/EUCAST_files/Breakpoint_tables/v_11.0_Breakpoint_Tables.pdf) and were spread on Mueller Hinton Agar plates (Bio-Rad, France) using sterile cotton swabs after which the MIC test strip was applied, and plates were incubated at 37°C for 18 h before the readings were recorded.

## 3. Results

### (a) pKSR100 has greater epidemiological success than pAPR100

As pKSR100 and pAPR100 had markedly different epidemiological success in the original study (conducted in the UK and France from 2008 to 2016) [27], we investigated whether the broader scientific research base and subsequent public health surveillance activity supported this discrepancy. In the original study, the pKSR100 carrying sublineage of *S. flexneri* 2a rapidly emerged as a dominant sublineage in a short space of time compared to the pAPR100 carrying sublineage even though the latter was circulating in the population prior to the acquisition of pKSR100 [27].

**Figure 1.**
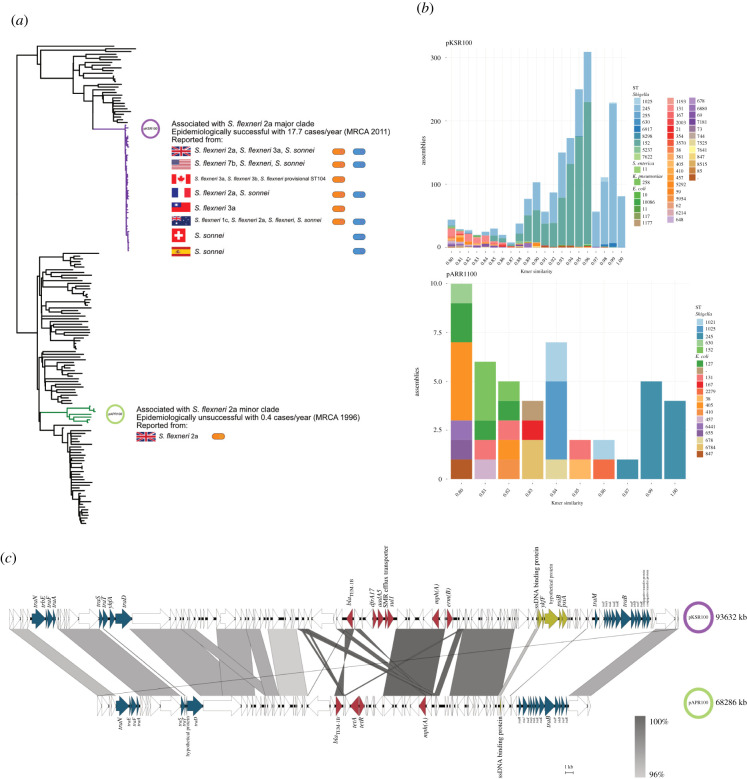
Relative epidemiological success and comparative genomics of pKSR100 and pAPR100. The disparate epidemiological success of pKSR100- and pAPR100- bearing clades in a cross section of 179 *Shigella* isolates from UK surveillance data between 2008 and 2014 [26]. The pKSR100-bearing major clade is highlighted in purple and the pARP100-bearing minor clade is highlighted in green (*a*). The pKSR100 associated major clade had a higher case rate despite a more recent MRCA (most recent common ancestor) compared to the lower case rate and older MRCA of the minor clade, highlighting the more rapid spread of the major clade through the UK. The information alongside the clades depict the disparate global and species distribution of other pKSR100 bacterial hosts being reported in multiple countries across multiple *Shigella* subtypes (as determined by BLASTn against the NCBI non-redundant database and literature review). pKSR100 and pKSR100-like plasmids have been detected in and reported from eight countries and in multiple *Shigella* subtypes. By contrast, pAPR100 has only been detected in *S. flexneri* 2a in the UK. (*b*) Species and sequence type distribution of genomes sharing kmer similarities of greater than 0.80 with pKSR100 or pAPR100 from across greater than 600 000 publicly available bacterial genomes (in the 661 K COBS data structure). (*c*) Comparison of the genetic content of both plasmids. Areas of synteny (using a cut-off of 95% BLAST identity) are shown intervening grey bars coloured according to the inlaid legend. Regions with variation between the two plasmids are broadly categorized into three main groups; (i) conjugation machinery related (blue), (ii) SOS response alleviation related (green) and (iii) AMR related (red). (Online version in colour.)

Since the original reporting of pKSR100 in Australia, Canada, France and the UK, further pKSR100 and pKSR100-like plasmids have been reported and/or deposited in the National Center for Biotechnology Information (NCBI) non-redundant database. Specifically, a BLASTn search returned 15 hits with over 95% query coverage and 95% identity from greater than or equal to 7 *Shigella* serotypes across four continents, continuing to drive national and global shigellosis dynamics [29,30,37–40] (figure 1*a*). Contrasting with the widespread dissemination of pKSR100, no BLAST hits with more than 87% query coverage for pAPR100 were returned, suggesting no distribution beyond its original detection in *S. flexneri* 2a in the UK. To extend the investigation of plasmid dissemination to unassembled genomes (such as those from routine public health surveillance activity), we screened both plasmids against the 661 K COBS data structure, which provides a snapshot of all bacterial genome data in the ENA as of November 2018 [41]. This search detected kmer similarity matches of over 0.80 of pKSR100 in 1926 publicly available bacterial genomes compared with only 46 bacterial genomes for pAPR100, further confirming the extensive dissemination of pKSR100. The genomes containing similarity matches for pKSR100 belonged to diverse bacterial hosts comprising 9 sequence types of *Shigella*, and 34 sequence types of *E. coli* and two other species (a *K. pneumoniae* ST258 and a *Salmonella enterica* ST11), while pAPR100 hits only came from 5 sequence types of *Shigella* and 13 sequence types of *E. coli* (figure 1*b*). Having established that pKSR100 had ongoing epidemiological success compared with pAPR100 through these analyses, we then set out to compare phenotypes of the two plasmids.

### (b) pKSR100 and pAPR100 broadly differ across three gene categories

To guide our phenotypic experiments, we first performed comparative genomic analysis of the plasmid sequences to identify areas of differing genetic content. The plasmids had a high degree of conservation between their genetic content (figure 1*c*, electronic supplementary material, file S2) with a total of 49 genes shared between both plasmids and 122 genes unique to each. However, the variances we observed with the gene content on both plasmids could be categorized into three broad functional categories; namely (i) conjugation machinery related genes, implying different conjugative abilities between pKSR100 and pAPR100 (ii) genes related to SOS response alleviation, indicating that perhaps pKSR100 conferred a fitness advantage related to stress responses and (iii) AMR genes, suggesting that perhaps the advantage of pKSR100 lay in conferring greater or broader resistance to antimicrobials (figure 1*c*). Thus, we then proceeded to measure and compare the phenotypic differences between these two plasmids with disparate epidemiological outcomes. The two sequences of the pKSR100 and pAPR100 variants used in this study have been uploaded to the NCBI under accession numbers CP090161 and CP090162, respectively.

### (c) pKSR100 is less conjugative than pAPR100 from its native donor

Although both plasmids carried the conjugation related *tra* genes and *trb* genes, we observed variations in the genes between the two plasmids (figure 1*c*). These variations at the nucleotide level were observed at less than 95% BLAST similarity between the two plasmid sequences. Given these discrepancies in conjugation related genes and the differential dissemination of the plasmids at an epidemiological level, we investigated the CE of pKSR100 and pAPR100 in shigellae and in model strain *E. coli* MG1655.

**Figure 2.**
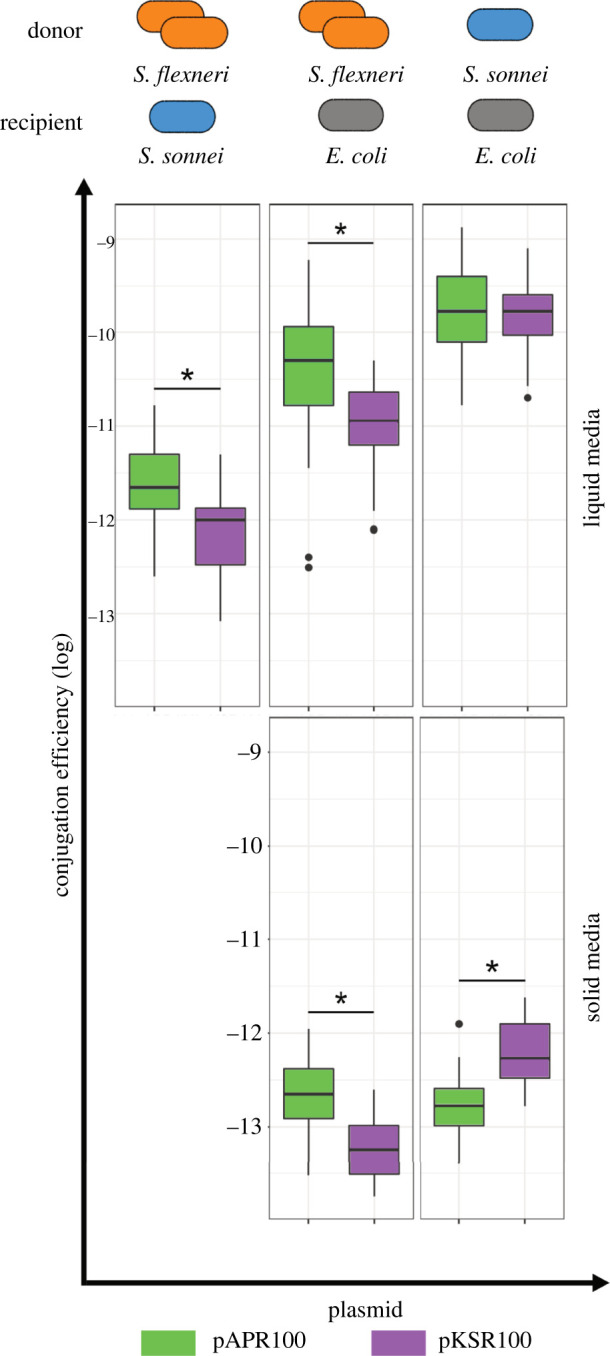
Conjugation efficiencies of pKSR100 and pAPR100 among different donors, recipients and media conditions. Different donor strains included native *S. flexneri* hosts (orange) and clinical isolate *S. sonnei* 216 as an isogenic donor (blue) while the recipient strains were either *E. coli* MG1655 (in grey) or *S. sonnei* 216 (in blue). Results for each plasmid are coloured according to the inlaid key. Results from liquid media are shown upper while solid is shown lower. Each box plot represents the combined results of all the time points used in LM models, where there are 12 replicates (four biological, three technical) for each time point. The asterisks denote significance as determined LMs. The *p*-values for each of the panels from left to right are as follows: top row, *p* = 0.043, *p* < 0.000; bottom row, *p* = 0.026, *p* = 0.025. Note: conjugations between *S. flexneri* donors and *S. sonnei* recipients are not shown here as no transconjugants were recovered until 3 h after mating, making it difficult to control for the growth rates of strains involved. Therefore, our main claims are based on liquid media conjugations where first transconjugants were recovered within 15 min of mating. (Online version in colour.)

Both pKSR100 and pAPR100 carried T4SS genes required for conjugation and were conjugative from *S. flexneri* to, and between, *E. coli* MG1655 and a clinical isolate of *S. sonnei* (figure 2). The *S. sonnei* clinical isolate we used, *S. sonnei* 216, from an MSM-associated sublineage that bore neither plasmid, but from the same collection of isolates as the native hosts of pKSR100 and pAPR100 [27] so represented a natural and relevant recipient. We measured CE across a number of variables including: from various donors (native and isogenic); to various recipients (*E. coli* and *S. sonnei* 216); in/on liquid and solid media; and across several time points (figure 2; electronic supplementary material, figure S2A).

Generally, pAPR100 had a better CE compared to pKSR100 from their native donors (i.e. the major and minor sublineages of *S. flexneri* 2a [27]) into either *S. sonnei* 216 or *E. coli* MG1655, regardless of media type (*post hoc* Tukey's tests on linear model (LM), *p* < 0.05 for all comparisons) (figure 2; electronic supplementary material, tables S5 and S4). However, these patterns varied across donors (liquid media LM hostpair : plasmid interaction *F*_2,87_ = 3.7, *p* < 0.029; solid media host : plasmid interaction *F*_1,28_ = 18.3, *p* < 0.001), such that in experiments with isogenic donors (*S. sonnei* 216 donation to *E. coli*) we detected no difference between the plasmids in liquid media (*post hoc* Tukey's test *p* = 1) and increased CE of pKSR100 versus pAPR100 on solid media (*p* = 0.025). This indicates that the CE differences between the plasmids observed with the native host donors may be due to the different donor backgrounds (electronic supplementary material, table S4). In support of the importance of donor effects, the isogenic *S. sonnei* donor facilitated a greater CE than the native *S. flexneri* donors (all relevant *post hoc* comparison *p* < 0.05) (electronic supplementary material, table S4). Recipient effects were also important as the *E. coli* recipient facilitated more efficient conjugation than *S. sonnei* (all relevant *post hoc* comparison *p* < 0.05) (electronic supplementary material, table S4).

### (d) pKSR100 has little fitness cost and alleviates the SOS response

**Figure 3.**
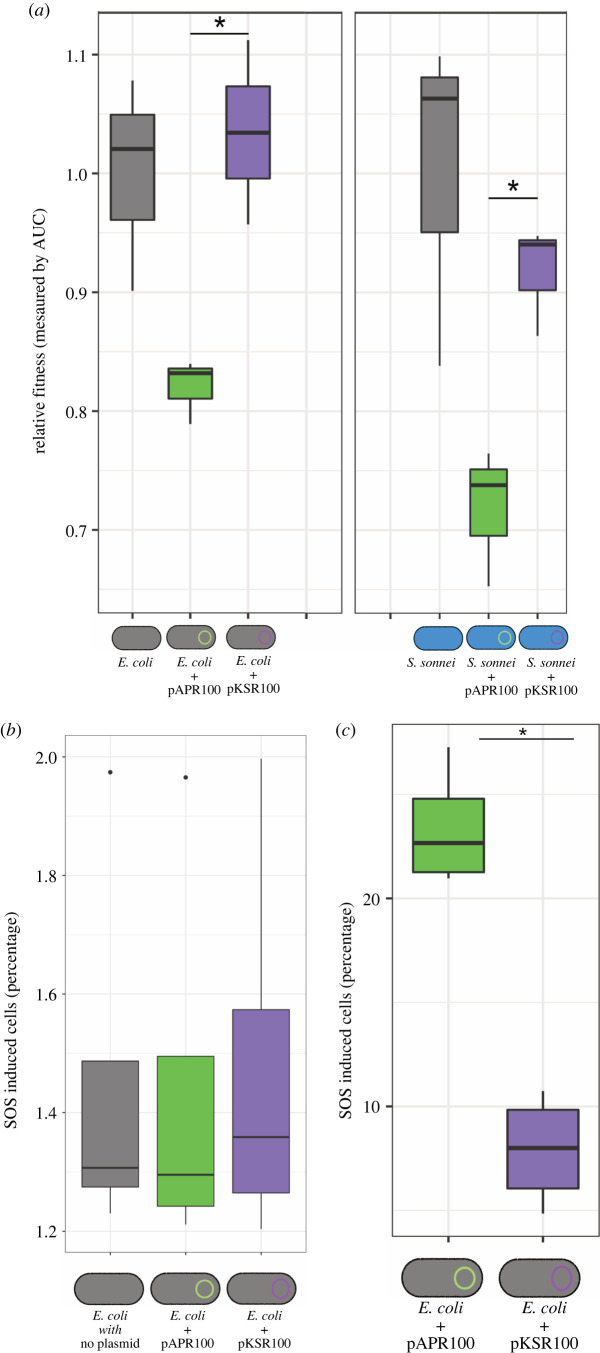
Relative fitness cost and SOS response induction during conjugation and antimicrobial exposure of pKSR100 and pAPR100. (*a*) Relative fitness of *E. coli* MG1655 (grey icons) and *S. sonnei* 216 (blue icons) carrying either pKSR100 (purple) or pAPR100 (green) compared to plasmid-free wild-type (grey bars in the graph). Asterisks denote significance where *p* = 0.01071 for *E. coli* strains and *p* = 0.009971 for *S. sonnei* strains as determined by two sample *t*-test. (*b*,*c*) SOS response levels by plasmid as a proportion of cells in which SOS is induced (measured by GFP expression using a reporter plasmid, see methods) during conjugation (*b*) and following a 2 h exposure to sub-inhibitory concentrations of ciprofloxacin (*c*). Individual box plots represent the median, range and IQR of four independent biological replicate data points adjusted to a negative control for each replicate (see methods, electronic supplementary material, figure S3). No statistically significant difference was observed between the two plasmids during conjugation (*p* = 0.7864), but there was a marked level of SOS response alleviation in cells carrying pKSR100 (*p* = 0.000237) as determined by a two-sample *t*-test. (Online version in colour.)

As plasmid burden on bacterial fitness is commonly investigated as a factor contributing to plasmid population dynamics, we investigated the fitness cost of plasmid carriage during growth. Comparing the growth of plasmid-bearing and plasmid-free strains revealed that pAPR100 imposed approximately 20% fitness cost in *E. coli* MG1655, whereas pKSR100 did not have a significant fitness cost based on the AUC measurements (figure 3*a*). Similar patterns were observed in *S. sonnei* 216, though unlike in *E. coli* we observed a small (approx. 6%) cost of pKSR100 in *S. sonnei*, suggesting that host factors also contribute to the overall relative fitness impact of plasmid carriage (figure 3*a*). Collectively, pAPR100 imposed an increased fitness cost relative to pKSR100 across the hosts.

The comparative genomic analysis revealed that pKSR100 contained a cluster of five genes including single stranded DNA binding protein gene, plasmid SOS inhibition A (*psiA*) and B (*psiB*) genes which were absent in pAPR100 (figure 1*c*; electronic supplementary material, figure S1A) and are known to alleviate the bacterial SOS response in *E. coli* [42]. A gene encoding for a hypothetical protein and an uncharacterized protein family (UPF) 0401 protein *ykfF* gene was also found in the same cluster though their roles in SOS response alleviation, if any, is unclear. The SOS response is often induced during conjugation and by environmental stressors and is known to be costly [43]. Given the disparate fitness costs between pKSR100 and pAPR100, we investigated whether there were differences in SOS response and alleviation attributable to the plasmids. Firstly, because of the differences between conjugation genes and the known activation of the SOS response during conjugation [35], we measured SOS response induction during conjugation anticipating that pAPR100 conjugation might elicit a greater SOS response in the recipients due to the absence of the gene cluster-containing *psiB*, and that pKSR100 (which has the *psiB* gene cluster) would alleviate the SOS response. However, we observed no statistically significant difference among SOS induction between the plasmids during conjugation when 100 000 events were counted (figure 3*b*) or when 1 million events were counted (data not shown), though both showed a slight increase in SOS relative to the negative control (figure 3*b*).

Because these plasmids derive from a patient community with high levels of antimicrobial use, we then sought to determine if either plasmid alleviated the SOS induction in response to sublethal concentrations of antibiotics. Specifically, we investigated the fluoroquinolone antibiotic ciprofloxacin, which is known to induce bacterial SOS response as a result of DNA mutagenesis stimulation in *E. coli* [44]; the recommended, and overwhelmingly administered, treatment for shigellosis in this patient community [45,46]; and an antimicrobial to which neither pKSR100 or pAPR100 confer resistance (see below and table 1). Our subsequent measuring of SOS induction among cells (carrying either pKSR100 or pAPR100) after a 2 h exposure to sub-inhibitory concentrations of ciprofloxacin revealed that cells harbouring pKSR100 were significantly better at alleviating the SOS induction than cells carrying pAPR100 (figure 3*c*). Thus cells harbouring pKSR100 were less likely to activate the costly SOS response following exposure of sublethal concentrations of an antimicrobial for which the plasmid conferred no resistance.

**Table 1.** The impact of pKSR100 and pAPR100 on antimicrobial resistance phenotypes.

					MIC values by plasmid and host^a^							
antimicrobial resistance			gene present in:		neither plasmid		with pKSR100			with pAPR100		
class	gene	antibiotic	pKSR100	pAPR100	*E. coli*	*S. sonnei* 216	*S. flexneri* (native)	*E. coli* + pKSR100	*S. sonnei* 216 + pKSR100	*S. flexneri* (native)	*E. coli* + pAPR100	*S. sonnei* 216 + pAPR100
macrolide	*mph(A)*	azithromycin	√	√	4	4	>256	**>256**	**>256**	48	**64/32**	**64**
macrolide	*erm(B)*	clindamycin	√		>256	48	>256	>256	**>256**	>256	>256	**>256**
sulfonamide	*sul1*	sulfamethoxazole	√		3	8	>1024	**12**	**>1024**	4	3	8
trimethoprim	*dfrA17*	trimethoprim	√		0.125	>32	>32	**>32**	>32	>32	0.125	>32
penicillins	*bla*_TEM1b/1a_	ampicillin	√	√	2	4	>256	**>256**	**>256**	>256	**>256**	**>256**
aminoglycoside	*aadA5*	streptomycin	√		1	16	48/64	**4**	24	128	1	16
tetracycline	*tetA*	tetracycline		√	0.75	0.75	0.75	0.75	0.75	64	**48**	**32**
fluoroquinolone		ciprofloxacin			0.006	0.004	0.006	0.006	0.004	0.004	0.006	0.006
cephalosporin		cefotaxime			0.016	<0.016	0.023	0.016	<0.016	0.016	0.016	<0.016

^a^MIC values in **bold** represent a change of four-fold or more in MIC when the relevant plasmid is present.

### (e) pKSR100 offers a greater range and magnitude of AMR

Owing to the importance of AMR in driving emergence of shigellosis among MSM populations, we also investigated the AMR phenotypes conferred by the two plasmids. Predictions of AMR genes identified six AMR genes in pKSR100, which accounted for 50% (6 of 12) of the total AMR genes in the host strain of *S. flexneri* 2a, while pAPR100 only had three AMR genes contributing 27% (3 of 11) to the total AMR gene content of its host *S. flexneri* 2a strain (electronic supplementary material, tables S2 and S3). Analysis of AMR genes present on the plasmids using ResFinder predicted that pKSR100 encoded resistance to sulphonamides, macrolides, trimethoprim, beta-lactams and aminoglycosides and that pAPR100 encoded resistance to macrolides, beta-lactams and tetracycline showing that the AMR genes of the plasmids differed both in the number and variety of drug classes they provided resistance against (table 1). Thus, both resistance plasmids conferred MDR and contained resistance genes relevant to transmission among MSM, but pKSR100 was predicted to confer broader-spectrum resistance.

To test the genotypic predictions of increased AMR conferred by the plasmids, we correlated our genotypic information with MIC phenotypes. The antimicrobials tested were based on the genotypic AMR predictions, and to differentiate plasmid and host factors, MICs were determined for the native hosts, and in both *E. coli* and *S. sonnei* 216 transconjugants of both plasmids compared to plasmid-free wild-types (table 1). The two plasmid-free hosts carried intrinsic resistance to three antibiotics (i.e. *E. coli* was resistant against clindamycin and *S. sonnei* 216 to trimethoprim and streptomycin). Collectively, however, the results demonstrated general agreement between the AMR genotype carried on plasmids and phenotype with a minimum fourfold increase in the MIC values compared to the controls when the AMR genes were present (table 1). Notably, however, pKSR100 conferred a higher level of resistance to azithromycin, a phenotype critical for driving circulation of shigellosis in this community at that time, by virtue of encoding the additional macrolide resistance gene *ermB* (table 1) [26,27].

In addition to those antimicrobials where resistance was anticipated to change based on genotypic prediction, we tested whether the plasmids conferred AMR against other clinically relevant antimicrobials; ciprofloxacin and cefotaxime. This was to ensure we were not missing relevant AMR conferred by potentially novel genes, or potentiating genes, and to ensure the robustness of our SOS induction results. As expected, neither plasmid altered the MIC values against these antimicrobials.

Collectively, these AMR results show that pKSR100 confers resistance to a more extensive range of antimicrobials, and a higher level of resistance to azithromycin, than pAPR100.

## 4. Discussion

Here, we investigated two AMR plasmids with different epidemiological outcomes from the same clinical niche to identify putative plasmid phenotypes associated with epidemiological success, using comparative genomics as a guide. And since the epidemiological fate of these plasmids had already been documented with regard to azithromycin resistance carried on these plasmids [26], our objective was to investigate the potential other factors that helped shape the observed epidemiological outcome of these plasmids beyond the fact that pKSR100 carried more AMR genes and conferred more AMR phenotypes. The spread and persistence of plasmids may depend on several plasmid and host associated factors such as the plasmid type, host range, conjugative capacity and fitness cost as well as the environments they are in [47–52]. Understanding the contributions of each of these factors is important in determining the fate of AMR plasmids found in clinical settings and predicting evolutionary trajectories of those plasmids [17]. In summary, our results identified that the epidemiologically successful pKSR100 conferred increased AMR, reduced fitness cost and reduced SOS response in the presence of sub-inhibitory concentrations of antibiotics, compared with the less successful pAPR100, despite pKSR100 having a lower CE in native hosts.

The epidemiologically prominent plasmid, pKSR100, carried more AMR genes than pAPR100. In addition to the AMR genes carried on the plasmid, the bacterial host also carried AMR genes on the chromosome (electronic supplementary material, tables S2 and S3). However, there were few discrepancies between the non-plasmid AMR gene content of the native hosts (only a *dfrA1* and additional *bla*TEM gene), and these did not result in phenotypic differences between the native hosts against the cognate antimicrobials (trimethoprim and ampicillin, respectively; table 1). Thus, it is unlikely that there was a relationship between the AMR genes present on the respective bacterial chromosomes (i.e. the *S. flexneri* 2a major and minor sublineages) and the spread of the plasmids. This is further evidenced by the remarkable onward global spread of pKSR100 from the setting in which it was originally described (figure 1). Thus, it is possible that the more extensive range of AMR and higher azithromycin resistance conferred by pKSR100 relative to pAPR100 was a key determining factor in the resulting epidemiological success and widespread dissemination of pKSR100. However, given that AMR genes frequently recombine between plasmids, and both plasmids already clearly encoded MDR, we are still faced with the question of why it was pKSR100 that acquired the broader AMR profile and went on to globally disseminate despite the comparative longevity of circulation of pAPR100 in the community. To address this, we considered further non-AMR factors guided by the comparative genomics, namely CE and fitness cost.

Conjugation is an efficient mechanism for plasmid dissemination and has been widely studied [1]. Theoretically, a higher conjugative capacity might result in a greater spread of plasmids. However, in our case, we observed that the globally widespread pKSR100 had a lower CE than pAPR100 from the native hosts. The observed differences in CE between pSKR100 and pAPR100 were mostly in the range of one order of magnitude (figure 2), but significant differences were observed for various combinations of donors and recipients, as well as various media conditions, indicating that, though modest, this effect is real. Furthermore, similar results were observed with different CE calculations (electronic supplementary material, figure S2B) adding to the persistency of our results. While the conjugative ability of plasmids can depend on donor or recipient strains' background as well as abiotic environmental factors, the lack of apparent importance of the CE exclusively for broader spread is consistent with a previous study examining the contribution of conjugation and other factors such as fitness effects towards plasmid spread in hospital settings [13]. However, we also observed that the differences of CE between the two plasmids diminished from an isogenic donor background, suggesting that donor factors are influencing CE in our model. This is not unexpected as the effect of donor and recipient backgrounds are known to affect the conjugative capacity of plasmids [53]. Hence, our results provide strong indications that the CE is dependent on donor, recipient and environmental conditions and that CE alone might not be an accurate measure of successful dissemination of a plasmid among bacterial populations in real world settings.

By contrast, the lack of a fitness cost imposed by pKSR100 may have been an important factor in perpetuating its epidemiological success. The fitness costs imposed by plasmids depend on the plasmid and host combination as well as their environmental factors along with gene conflicts [47,54] and, previous work has demonstrated that fitness cost measured in a laboratory setting might not translate to success in the natural environment [23]. We addressed host variability by measuring the fitness cost imposed by our plasmids in multiple backgrounds, and the broader database analysis (figure 1; electronic supplementary material, figure S1B) indicated a similar host range for the two plasmids and their close relatives. While our results may not relate exactly to the fitness cost imposed by these plasmids on their natural hosts, it helps us understand the effects that fitness costs may have on transmission through other residents in their natural gut microbe community. Our observation of the unsuccessful pAPR100 having a greater fitness cost is consistent with fitness costs affecting plasmid fates in microbial populations, and suggests that fundamental plasmid properties can contribute to epidemiological outcomes, specifically in a community-transmitting pathogen in our case.

Given the difference in SOS alleviation gene content between the two plasmids, we also examined the impact of the two plasmids on the induction of the costly SOS response. Since plasmids move as single stranded DNA molecules to recipient cells during conjugation, the possible induction of the SOS response may act as an influencing factor for the dissemination of plasmids. The pKSR100-related plasmid R100 had been shown to induce the SOS response when conjugated into *Vibrio cholerae* recipient (alleviated by the *psiB* gene cluster [55,56]). Hence, finding no difference in SOS induction during conjugation with the *psiB* cluster-containing pKSR100 and pAPR100 (which does not contain the cluster) in *E. coli*, indicates that SOS inductions by plasmids could be host dependent. However, as sub-inhibitory concentrations of ciprofloxacin have been demonstrated to induce the SOS response [57] and this is a highly relevant exposure for our system (as ciprofloxacin is a recommended treatment for shigellosis and other STIs circulating among MSM [45]) our finding that pKSR100 protected against the induction of the SOS response during exposure to sub-inhibitory concentrations of a clinically relevant antimicrobial supports the notion that an SOS response alleviation phenotype may contribute to AMR emergence in an epidemiological setting of high antimicrobial use.

The results of our study are consistent with the theory that, alongside AMR, plasmid-associated non-AMR phenotypes may play a crucial role in facilitating the dissemination of AMR plasmids. Although our study used only two plasmids from a single model system, the plasmids we used were important examples drawn from an established pathogen and real world epidemiological scenario (capturing complex networks in their natural habitats). Thus, our findings may help guide future studies towards the development of universal ground truths regarding important plasmid phenotypes for AMR emergence and establishment within the dynamic and problematic *Enterobacteriaceae*. We also mitigated the possibility that phenotypic findings may have been dependent on non-plasmid factors by varying the host and environmental conditions, which will vary in the natural habitat. However, our study raises further questions, particularly concerning the molecular mechanisms behind the disparate epidemiological outcomes of the two plasmids. Interestingly, though we could generate strains carrying both pKSR100 and pAPR100 in the laboratory (electronic supplementary material), this pairing has never been detected in any of our collection of approximately 179 epidemiological isolates. The separation of these two plasmids could be due to specific evolutionary conflicts and deleterious interactions of the two plasmids in their natural hosts. Further investigations into the evolutionary consequences of harbouring both plasmids is another subject for future research. In addition to that, our observations on SOS response alleviation after exposure to ciprofloxacin in the presence of pKSR100 could also be taken further to evaluate the long-term survival of ciprofloxacin exposure as another future research objective. Together, our data demonstrate that working backwards from an established epidemiological scenario is a valuable approach for identifying plasmid-associated phenotypes that contribute to the epidemiological trajectory of AMR, and perhaps one day could be turned prospectively to aid prediction and prevention of AMR emergence in future surveillance.

## Data Availability

Plasmid sequences used are publicly available in NCBI database and the accession numbers are provided in the main text. Raw data for used for the linear modelling and electronic supplementary material, file S2 are deposited in the Dryad Digital Repository (doi:10.5061/dryad.sxksn0363) [58]. Electronic supplementary material is available online [59].
